# BRCA1-A and BRISC: Multifunctional Molecular Machines for Ubiquitin Signaling

**DOI:** 10.3390/biom10111503

**Published:** 2020-10-31

**Authors:** Julius Rabl

**Affiliations:** Cryo-EM Knowledge Hub, ETH Zürich, Otto-Stern-Weg 3, HPM C51, 8093 Zürich, Switzerland; jrabl@ethz.ch; Tel.: +41-44-633-2099

**Keywords:** deubiquitination, BRCA1, DNA repair, immune regulation, BRCC36, SHMT2, RAP80, ubiquitin, SUMO

## Abstract

The K63-linkage specific deubiquitinase BRCC36 forms the core of two multi-subunit deubiquitination complexes: BRCA1-A and BRISC. BRCA1-A is recruited to DNA repair *foci*, edits ubiquitin signals on chromatin, and sequesters BRCA1 away from the site of damage, suppressing homologous recombination by limiting resection. BRISC forms a complex with metabolic enzyme SHMT2 and regulates the immune response, mitosis, and hematopoiesis. Almost two decades of research have revealed how BRCA1-A and BRISC use the same core of subunits to perform very distinct biological tasks.

## 1. Introduction

### 1.1. K63-Linked Ubiquitination

A fundamental mechanism underlying cellular signalling is the specific, covalent modification of proteins. Sophisticated machinery has evolved to specifically form or break isopeptide bonds between the ε-amino group of lysines and the carboxyl terminus of the small, highly conserved protein ubiquitin [1]. Ubiquitin signaling is extensive and essential for eukaryotic life and similar systems are even present in prokaryotes [2,3]. Ubiquitination sites have been identified on thousands of proteins and the patterns change dynamically in response to physiological changes [4].

Ubiquitin molecules carry seven lysines on their surface, at positions K6, K11, K27, K29, K33, K48, and K63. The ε-amino group of each lysine as well as the amino terminus of the protein can engage in an isopeptide bond with the carboxyl terminus of another ubiquitin, forming a ubiquitin chain [1]. In this fashion, eight different types of unbranched ubiquitin chains can be formed. Ubiquitin chains differ structurally depending on linkage, from the open, accessible linear or K63-linked chains, to the closed and clustered K48-linked chains, and they are processed by specialized, linkage-sensing machinery that uses them in independent signaling pathways [5,6,7,8].

K48-linked ubiquitin chains are most common and target proteins for degradation via the ubiquitin proteasome pathway [9]. K63-linked ubiquitination, the second most common ubiquitin chain type, carries signals essential for DNA repair, the stress and immune response, and membrane protein homeostasis [10,11].

### 1.2. Deubiquitination Complexes

The enzyme inventory of human cells contains ∼100 de-ubiquitinases (DUBs), enzymes that specifically cleave the isopeptide bond between the carboxy-terminus of ubiquitin and either the N-terminus or the ε-amino group of a lysine of another protein. DUBs show a wide spectrum of activity and linkage specificity. While many DUBs are apparently monomeric, unregulated, and functionally rather promiscuous, some DUBs show high substrate specificity and some degree of regulation [12,13]. DUBs incorporated into large molecular machines, such as the proteasome lid, can acquire additional specificity and regulation from other subunits in the complex [14].

### 1.3. Discovery of BRCA1-A and BRISC

Interaction between DNA repair associated ubiquitin E3 ligase BRCA1 and protease BRCC36 was first reported in 2003 [15]. In the same report, BRE was already identified as member of the complex. Four years later, in 2007, three groups reported simultaneously that BRCA1 forms a complex with RAP80, a DNA-repair associated protein, and that this complex targets BRCA1 specifically to DNA repair *foci* [16,17,18]. Interaction between RAP80 and BRCA1 was found to be mediated by a new protein subunit of the complex, named ABRAXAS, that binds to BRCA1 when phosphorylated [18]. ABRAXAS was found to have a homolog, which was named ABRO1 [18]. In 2009, a complex of BRCC36, BRE, a subunit initially called HSPC142, and ABRO1 was purified from HeLa cells and found to have high K63-specific DUB activity [19]. At the same time, HSPC142 was identified as essential component of the BRCA1-binding complex and renamed MERIT40 (or, less frequently used, NBA1) [20,21,22]. In 2011 it became clear that BRCC36 is the catalytically active subunit of two distinct DUB complexes, BRCA1-A and BRISC, that share three subunits, but are targeted to different compartments and carry out different physiological tasks [23]. Structure determination of the ancestral insect subcomplex KIAA0157-BRCC36 [24], mouse BRCA1-A complex [25] and human BRISC-SHMT2 complex [25,26] finally allowed to rationalize almost two decades of biochemical and cellular data and laid the foundation for understanding BRCA1-A and BRISC function on the molecular level.

### 1.4. Evolution of BRCA1-A and BRISC

Homologs of BRCC36 have been identified in plants and are involved in DNA repair [27]. A complex containing BRCC36, scaffold protein KIAA0157, BRE, and MERIT40 is present in insects and efficiently degrades K63-linked chains [24]. The subfunctionalization of ancestral scaffold KIAA0157 into ABRAXAS and ABRO1 after a whole genome duplication prior to the development of cyclostomes established BRCA1-A and BRISC as separate complexes with diverging function; they are found in all vertebrates (diagram in Figure S1C of [25]). All human tissues express subunits of BRCA1-A and BRISC [28,29].

## 2. Subunits and Complex Assembly

### 2.1. Nomenclature

**Figure 1:**
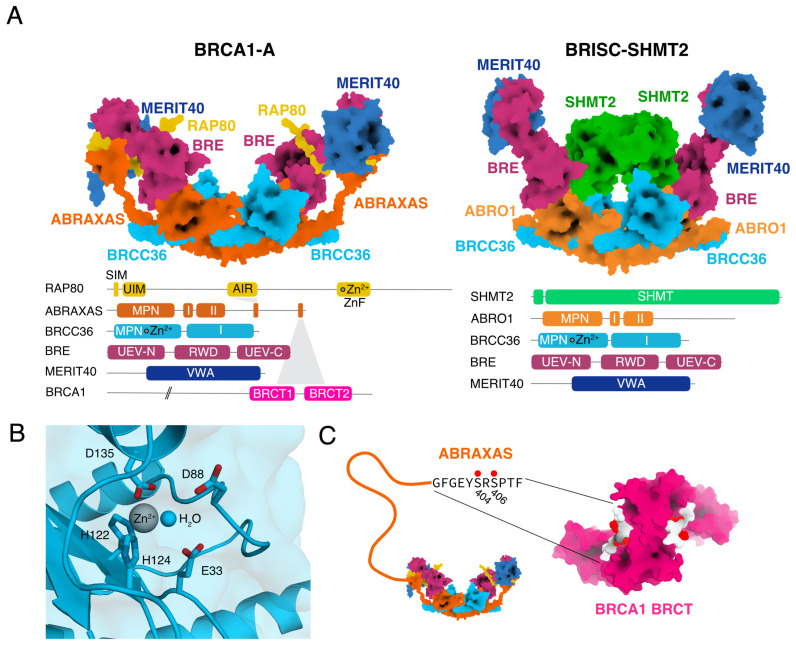
(**A**) Architecture of BRCA1-A and BRISC-SHMT2α complex. Schematic representation of protein subunits and binding partners (BRCA1-A and BRISC-SHMT2α structure: [25]). (**B**) Detailed view of the active site of BRCC36. The side chains of catalytic residues E33, D88, H122, H124, and D135 are shown. The zinc atom at the active center is shown in silver. A catalytic water molecule is shown as a blue sphere (BRCA1-A structure: [25]). (**C**) The BRCA1 BRCT dimer binds to the phosphorylated C-terminus of ABRAXAS. The flexible C-terminus of ABRAXAS is depicted schematically and the conserved BRCT binding sequence of ABRAXAS is shown. The phosphorylation sites at S404 and S406 are indicated with red dots. The BRCA1 BRCT domain dimer bound to the phosphorylated ABRAXAS C-terminus is shown in surface representation with the BRCA1 shown in pink and the ABRAXAS C-terminal tail in white with phosphorylation sites shown in red (BRCA1 BRCT structure: [30], BRCA1-A structure: [25]).

This section provides an overview of the protein subunits in BRCA1-A and BRISC and describes how they assemble to form the functional complexes. Nomenclature and definition of the complexes has evolved since the initial discovery of an association between BRCC36 and BRCA1. While a new and more consistent nomenclature has been employed recently [26], this review uses the legacy nomenclature and complex definition used in the majority of the literature. The BRCA1-A complex consists of ABRAXAS, BRCC36, BRE, MERIT40, and RAP80. BRCA1-A binds BRCA1 to form the BRCA1-A-BRCA1 complex (Figure 1A). BRISC consists of ABRO1, BRCC36, BRE, and MERIT40 and it binds SHMT2 to form the BRISC-SHMT2 complex (Figure 1A). All residue numbers are given for the human proteins unless stated otherwise (Table 1).

### 2.2. BRCC36

BRCC36 contains an N-terminal JAMM/MPN${}^{+}$ domain, followed by a coiled-coil domain [17,31]. The JAMM/MPN${}^{+}$ metalloprotease domain in BRCC36 specifically cleaves isopeptide bonds between the lysine ϵ-amino group and the C-terminal carboxyl group of ubiquitin with a specificity for K63-linked ubiquitin chains [32]. A zinc atom forms the active center of BRCC36, coordinated by two histidines (H122 and H124), an aspartate (D135), and one molecule of water [24,25,26] (Figure 1B). A nucleophilic attack on the isopeptide bond by the zinc-activated water molecule results in a tetrahedral intermediate followed by isopeptide bond cleavage and simultaneous release of the reaction products, followed by enzyme regeneration [33]. In BRCA1-A and BRISC complex, only BRCC36 is enzymatically active, the other subunits serve structural and regulatory purposes.

### 2.3. ABRAXAS

ABRAXAS contains an N-terminal JAMM/MPN${}^{-}$ domain, which is homologous to the JAMM/MPN${}^{+}$ domain, but enzymatically inactive due to loss of the zinc binding site [18,31]. Inactive as a DUB, the JAMM/MPN${}^{-}$ domain of ABRAXAS binds to the JAMM/MPN${}^{+}$ domain of BRCC36 and activates it [24]. A similar arrangement of an active JAMM/MPN${}^{+}$ isopeptidase that is activated by the JAMM/MPN${}^{-}$ domain of a scaffold protein is found in the proteasome lid and the COP9 signalosome [34,35]. The JAMM/MPN${}^{-}$ domain of ABRAXAS is followed by a coiled-coil domain and a long, unstructured C-terminus, which terminates in a twin phosphorylation site (S404, S406) that is recognized by the BRCT domain of BRCA1 [18,30] (Figure 1A). ABRAXAS carries a nuclear import signal that is essential for the nuclear localization of the entire BRCA1-A-BRCA1 complex [36].

### 2.4. ABRO1

Like ABRAXAS, ABRO1 contains an N-terminal JAMM/MPN${}^{-}$ domain that is not enzymatically active and serves as activator and scaffold [23]. The JAMM/MPN${}^{-}$ domain is followed by a long C-terminal unstructured tail that contains a phosphorylation site at Y377, which is bound by the SH2 domain of LNK [23,37].

### 2.5. BRE

**Figure 2:**
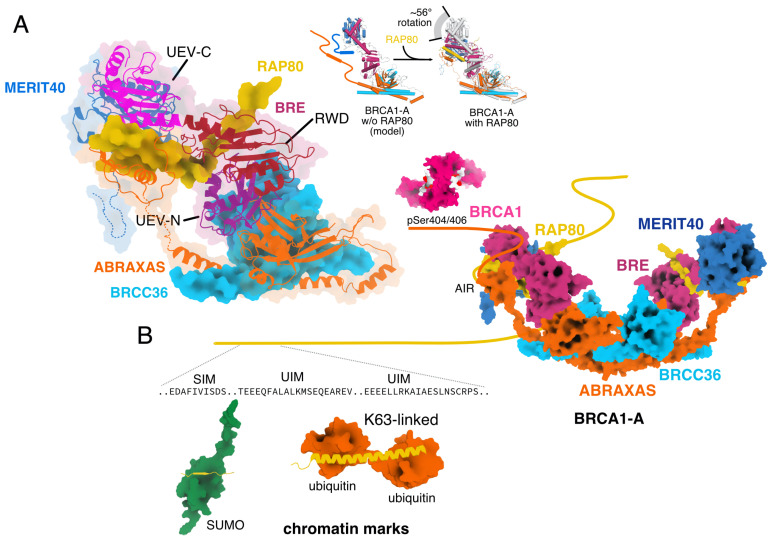
(**A**) Integration of RAP80 into the BRCA1-A complex. RAP80 and BRCC36 are shown as surface representation, ABRAXAS, BRE and MERIT40 are shown as cartoon. Integration of RAP80 into BRCA1-A induces a substantial conformation change in BRE, which results in a rotation of ∼56${}^{\circ }$ towards the coiled coils at the base of the complex (BRCA1-A and BRISC-SHMT2α structure: [25]). (**B**) Schematic representation of BRCA1-A binding to chromatin marks. The SIM and twin UIM domains at the N-terminus of RAP80 are tethered by a long flexible linker to BRCA1-A. BRCA1 binds to the C-terminus of ABRAXAS (BRCA1-A structure: [25], BRCA1 BRCT domain with bound ABRAXAS phosphopeptide structure: [30], RAP80 SIM-bound ubiquitin structure: [41], RAP80 UIM-bound K63-linked ubiquitin structure: [42]).

BRE contains three domains: an N-terminal (UEV-N) and a C-terminal UEV domain (UEV-C), and between them a central RWD domain [25,26]. UEV and RWD domains are structurally related to E2 enzymes and mediate interactions between proteins, often including ubiquitin [38,39]. The UEV-N domain of BRE binds ABRAXAS/ABRO1, while the UEV-C domain binds MERIT40 [25]. The interaction surface between the UEV-N and the RWD domain is considerably larger than the interaction between the RWD and the UEV-N domains, leaving a channel that in BRCA1-A complex is filled by the AIR domain of RAP80 [25] (Figure 2A). In absence of RAP80, this channel functions as a flexible hinge, allowing considerable movement between the UEV-N/RWD and the UEV-C domain of BRE, as evident in the structures of BRISC-SHMT2 complex [25,26]. When not occupied by RAP80, the cleft in BRE mediates dimerization of two complexes to form a dimer of dimers [25,26,40].

BRE binds ubiquitin: a soluble complex of BRE and MERIT40 (BRE requires MERIT40 for solubility) binds ubiquitin with low micromolar affinity (K${}_{d}$ ∼ 20 μM), substantially tighter than the affinitiy measured for MERIT40 alone [25].

### 2.6. MERIT40

MERIT40 consists of an unstructured N-terminus and a C-terminal VWA domain [22,25]. VWA domains are similar in function to UEV domains: they mediate protein-protein interactions, including interactions with ubiquitin [43]. The unstructured N-terminus contains a tankyrase binding site and is weakly parsylated in vivo [44]. MERIT40 is the only subunit of BRCA1-A or BRISC that can be expressed in full-length and purified in isolation and binds ubiquitin with high micromolar affinity [25,45].

### 2.7. RAP80

RAP80 is an extended, largely unstructured protein. The N-terminus contains a SIM domain (residues 40–43) that binds SUMO with micromolar affinity (K${}_{d}$∼ 200 μM) [46,47,48]. Binding affinity of the SIM domain is regulated by phosphorylation of two adjacent serines (S44 and S46), which substantially increases SUMO binding affinity (K${}_{d}$∼ 9 μM) [41].

Two UIM domains (UIM1: residues 80–96, UIM2: residues 104–120) are located close to the SIM domain. Both UIMs are helical and they are connected by a rigid helix so that simultaneous binding by two ubiquitin moieties is only possible when they are K63-linked [42]. RAP80 binds K63-linked di-ubiquitin with low micromolar affinity (K${}_{d}$∼ 17 μM) [47], but the SIM and UIM domains of RAP80 bind mixed chains of SUMO and K-63-linked ubiquitin non-cooperatively with nanomolar affinity (K${}_{d}$ ∼ 200 nM) [47,48] (Figure 2B).

The central AIR domain of RAP80 (residues 272–330) mediates integration into the BRCA1-A complex. RAP80 AIR binds MERIT40, fills the channel between the RWD and UEV-C domains of BRE, and is embedded into BRCA1-A by a short domain of the ABRAXAS C-terminus [25].

### 2.8. Assembly of BRCA1-A and BRISC Complex

BRCA1-A complex is a dimer of pentamers with C2 symmetry [25] (Figure 1A). Each pentamer contains one copy of ABRAXAS, BRCC36, BRE, MERIT40, and RAP80. ABRAXAS and BRCC36 form an MPN dimer that is structurally different from the MPN dimers found in the proteasome lid and COP9 signalosome complex [24,25]. Unlike RPN8 and RPN11 in the proteasome lid or CSN5 and CSN6 in the COP9 signalosome, the interaction between ABRAXAS and BRCC36 is mediated by β-sheets [24,25]. The UEV-N domain of BRE binds to ABRAXAS and the UEV-C domain binds MERIT40 [25] (Figure 2A). The AIR domain of RAP80 binds in the channel between the RWD and UEV-C domains of BRE, and contacts MERIT40 [25] (Figure 2A). The unstructured C-terminus of ABRAXAS binds on top of RAP80 and embeds RAP80 into the BRCA1-A complex [25] (Figure 2A). Two pentamers dimerize to form a complete, arc-shaped BRCA1-A complex [24,25,40] (Figure 1A). The dimerization is mediated by the coiled coil domains of BRCC36 and ABRAXAS, which form a helical bundle, similar to the assembly principle of the COP9 signalosome [24,25,34]. Cross-bracing by the linkers between the JAMM/MPN${}^{-}$ domains and the coiled-coil domain of ABRAXAS renders non-destructive, reversible separation of the dimers unlikely [25] and loss of RAP80 in vivo leads to loss of the functional BRCA1-A complex [49,50]. Fully assembled BRCA1-A complex does not form the “super-tetrameric” complex observed when BRCA1-A is expressed in absence of RAP80 since the interaction surface on BRE is saturated [25,40].

BRISC complex forms a dimer of tetramers with C2 symmetry [25,26] (Figure 1A). Each tetramer contains one subunit of ABRO1, BRCC36, BRE, and MERIT40 [25,26]. The JAMM/MPN domains of ABRO1 and BRCC36 are found in an identical arrangement to BRCA1-A and the connections mediating the dimerization are identical to what is observed in BRCA1-A [25]. ABRO1 is bound by the UEV-N domain of BRE, which binds MERIT40 with the UEV-C domain [25]. Due to the absence of RAP80, BRE is not structurally locked in by the AIR domain bridging between the RWD and UEV-C domain (Figure 2A), leaving BRE flexible and the “super-tetramerization surface” exposed [25,26]. BRISC arcs form interlocking dimers driven by a weak interaction between BRE and BRCC36, but this “super-tetramerization” is easily disrupted by ubiquitin substrate or SHMT2 binding [25].

## 3. BRCA1-A Suppresses Homologous Recombination

### 3.1. Ubiquitin Marks Recruit BRCA1-A

What is the function of BRCA1-A in DNA repair? DNA double-strand breaks are highly toxic lesions that are immediately recognized by sophisticated cellular repair machinery. If a cell experiences DNA damage during the S or G2 phase, the available sister chromatid will serve as template for a loss-free repair via the homologous recombination (HR) pathway. Outside of S and G2 phase, the cell uses non-homologous end joining (NHEJ), where it removes the overhangs and re-ligates the DNA ends [51]. As DNA repair has the potential to induce genomic instability, pathway choice is tightly controlled [52].

**Figure 3:**
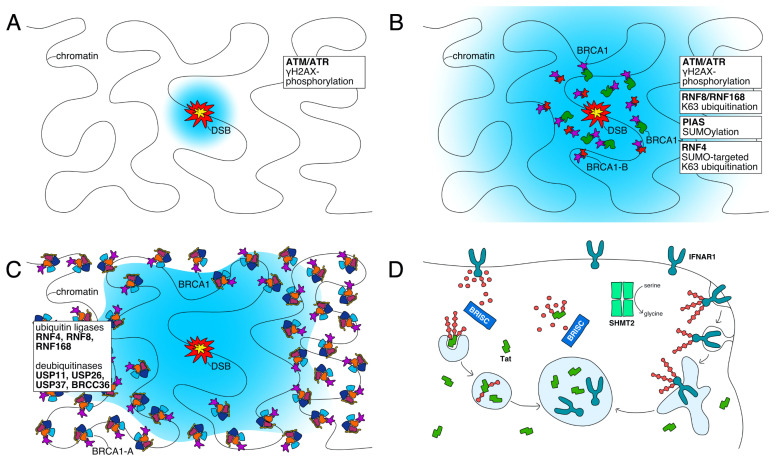
Schematic representation of DNA double-strand break repair associated signaling events (**A**–**C**) and interferon and Tat degradation control by BRISC (**D**). (**A**) A DNA double-strand break (red, center) has been detected, ARM/ATR marks chromatin surrounding the site of damage (blue). (**B**) Signaling by ubiquitin ligases, sumo ligases, and SUMO-targeted ubiquitin ligases marks chromatin surrounding the site of damage (blue) while BRCA1 is recruited to the site of damage where it engages in resection-promoting complexes. (**C**) DUBs including BRCC36 edit the chromatin marks (blue) and limit their spread. BRCA1 is sequestered to the periphery of the DNA repair focus and withdrawn from the site of damage. (**D**) BRISC degrades K63-linked chains that serve as lysosomal degradation signals from membrane receptor IFNAR1 and soluble viral protein Tat, increasing their stability and concentration.

Ubiquitin and SUMO signals define the DNA repair *focus*, govern the recruitment of DNA repair enzymes, and control their activitiy [53,54]. DNA damage activates ATM, which phosphorylates histone H2AX [55] (Figure 3A). The γH2AX signal is in turn recognized by MDC1, which recruits ubiquitin E3 ligases RNF8 and RNF168 [56]. RNF168 primes ubiquitin chain generation by monoubiquitination of K13 and K15 of histone H2A/H2AX and deposits K63-linked ubiquitin chains on histone H1 [57,58,59]. Primed by RNF168 monoubiquitination of H2A/H2AX, RNF8 extends the monoubiquitin marks with K63-linked chains [59]. At the same time, SUMO ligases PIAS1 and PIAS4 synthesize SUMO chains on various enzymes at DNA repair *foci* and these chains activate the SUMO-targeted ubiquitin ligase RNF4, which extends them as mixed SUMO-K63-linked ubiquitin chains [57,60,61] (Figure 3B). As a result of these processes, micron sized zones containing kilobases of chromatin around the site of damage accumulate marks that include K63-linked ubiquitin chains mixed with SUMO [60]. The N-terminus of BRCA1-A subunit RAP80 carries a SIM-UIM domain that binds SUMO and ubiquitin with high affinity [47,48]. With SUMO and ubiquitin chains accumulating at DNA repair *foci*, the BRCA1-A complex with bound BRCA1 is recruited to the modified chromatin region [16,17,18] (Figure 2B).

### 3.2. BRCA1 Sequestration by BRCA1-A

Initially recruited both to the site of damage as well as to the surrounding chromatin, BRCA1 promotes in a POH1-dependent process the redistribution of 53BP1, RAP80 and ubiquitin chains towards the periphery of the DNA repair *foci*, leaving the area immediately at the site of damage depleted [62]. BRCA1-A binds BRCA1 with high affinity and withdraws it from the site of damage to the periphery where it remains sequestered [63] (Figure 3C). The patterns that recruit RAP80 are reinforced by recruitment of E3 ubiquitin ligases RNF8 and RNF168 to the periphery [62,63]. Sequestration of BRCA1 in the periphery, away from the DNA damage site suppresses HR and steers the cell toward repair by NHEJ, while loss of RAP80 leads to concentration of BRCA1 at the site of damage, excessive resection, hyperactive HR and finally genomic instability [64].

### 3.3. Ubiquitin Processing at DNA Repair Foci

BRCC36, when incorporated into the BRCA1-A complex, is an active K63-linkage specific DUB that is targeted by RAP80 to K63-linked ubiquitin chains in DNA repair *foci* [17,25,42,47,48]. With recruitment relying on RAP80 recognition of K63-linked ubiquitin chains, accumulation of BRCA1-A at DNA repair *foci* would be expected to contribute to their degradation. An active-site-dead mutant of BRCC36 that still forms the entire BRCA1-A complex including RAP80 and BRCA1 does result in increased ubiquitination at histone γH2AX after irradiation [65]. Yet, deubiquitination by BRCC36 apparently serves roles beyond simply removing DNA damage signals from chromatin: supplanting BRCC36 with an active-site-dead mutant in human cells disrupts IRIF formation of RAP80 and ABRAXAS and promotes increased resection. Degradation of K63-linked chains by BRCC36 reinforces recruitment of BRCA1-A [66], suggesting that BRCC36 edits rather than degrades chromatin signals.

## 4. BRISC Functions in Immune Response, Mitosis and Hematopoiesis

### 4.1. Interferon Response Control by BRISC-SHMT2α

Sharing the same DUB subunit with BRCA1-A, BRISC functions in starkly different pathways. Membrane proteins are degraded via the endosomal-lysosomal pathway: Targets for degradation are marked with K63-linked ubiquitin chains, which are recognized by the endosomal sorting complexes, which in turn deliver the target to the lysosome where it is degraded. The abundance of membrane receptors is regulated by a tug-of-war between E3 ligases that deposit degradation signals and DUBs that remove the degradation signals and thereby rescue the membrane proteins from degradation [11]. Similar to other DUBs that counteract degradation of specific membrane proteins (e.g., AMSH rescuing EGFR [11]) BRISC counteracts ubiquitination of IFNAR1 and the HIV-1 Tat protein [67,68].

In response to viral challenges, interferons are released [69]. These small signaling proteins belong to the class of cytokines and cannot cross the cell membrane, but instead bind specific receptors on the cellular surface that transmit the signal by a signal cascade until it ultimately reaches the nucleus and activates transcription [69]. While interferon signaling protects against challenges from viral infection, prolonged response leads to inflammation-induced tissue damage, necessitating a mechanism that tunes how receptive the cell is to interferon stimulation [70].

Type 1 interferon IFN-β binds receptor IFNAR1 in a nanomolar affinity complex that induces gene expression by complex formation with IFNAR2 and subsequent activation of Jak-STAT as well as via an IFNAR2-independent mechanism [71]. Cellular response to IFN-β is regulated by modulation of the abundance of the IFNAR1 receptor on the cell surface: Casein kinase CK1α phosphorylates serine S535 of IFNAR1, which promotes ubiquitination of three lysines by E3 ubiquitin ligase SCF${}^{\beta -Trcp}$ [72,73]. Polyubiquitination with both K48- and K63-linked chains exposes a linear endocytic motif, promotes binding to adaptin which in turn leads to endocytosis and lysosomal degradation of the receptor [74]. Ubiquitination of IFNAR1 codependently recruits SHMT2 and BRISC [68]. BRISC deconjugates the ubiquitin chains off IFNAR1, reducing the extent to which the receptor is removed from the cell surface by endocytosis [68] (Figure 3D). The activity of BRISC counteracts the ubiquitination and degradation of IFNAR1 and promotes the cellular response to interferon 1: Specific disruption of BRISC scaffold subunit ABRO1 in serveral systems reduced the scale of the interferon response and limited tissue damage caused by septic shock [68].

### 4.2. BRISC Counteracts Degradation of HIV-1 Tat

HIV-1 virus encodes Tat, a soluble protein capable of penetrating the cell wall, which, activated by ubiquitination, promotes transcription of viral genes and interferes with the human immune response [75]. BRISC in concert with SHMT2 binds to Tat and deconjugates the K63-linked ubiquitin chains, increasing the lifetime of the protein, but deactivating it [67] (Figure 3D).

### 4.3. BRISC Functions in Mitosis

BRISC binds to microtubules and regulates the assembly of mitotic spindle components by controlling the K63-linked ubiquitination of NuMA [76]. Nuclear enzyme tankyrase is recruited to telomeres, stabilized by K63-linked ubiquitin chains synthesized by RNF8, and controls resolution of telomere cohesion [77]. Dissolution of the nuclear envelope during mitosis allows BRISC complex access to and degradation of tankyrase bound K63-linked ubiquitin chains, which results in dissociation from telomeres and signals that telomere cohesion is resolved [77].

### 4.4. BRISC Controls JAK2 in Hematopoiesis

Hematopoietic stem cell expansion is regulated by K63-linked ubiquitin signaling [37]. Stimulation by thrombopoietin induces K63-linked ubiquitination of JAK2. BRISC is recruited to JAK2 by LNK, binding the SH2 domain with a phosphorylated tyrosine (Y377) on the C-terminal tail of ABRO1 [37]. BRISC deubiquitinates JAK2, counteracting JAK2 signaling by rendering it prone to degradation [37].

## 5. Specificity and Regulation of BRCC36

### 5.1. Activation by Assembly

BRCC36, the shared active subunit, is at the center of BRCA1-A and BRISC activity. How is BRCC36 regulated? Are there differences in regulation between the complexes? Purified BRCC36 is inactive, but gains robust DUB activity when integrated into BRCA1-A or BRISC complex [15,24,78]. BRCC36 in isolation shows substantial disorder in the E-loop region, which moves the catalytic glutamate (E33) away from the active site and renders the enzyme inactive [24]. ABRAXAS and ABRO1, which activate BRCC36 when forming a complex, contain MPN domains, but lack active site residues and are not enzymatically active [18,78]. BRCC36 is activated by the same mechanism in the ancestral KIAA0157-BRCC36 complex, in mammalian BRCA1-A, and mammalian BRISC complexes: assembly of the BRCC36 heterodimer with the scaffold domain positions an arginine of the scaffold domain (N177 in *Camponotus floridanus* KIAA0157, N164 in ABRO1, and N170 ABRAXAS) near a glutamate (E30), structuring the E-loop [24,25]. Structuring of the E-loop moves the catalytic glutamate towards the zinc atom and activates BRCC36 [24,25]. While inactivity of the isolated MPN+ subunit and activation upon dimerization with a MPN- scaffold subunit was observed with other MPN+ isopeptidases, such as RPN11 and CSN5, the mechanism described above is unique to BRCC36 [24,25,34,35,79].

### 5.2. Linkage Specificity

BRCC36 is strictly specific for K63-linked ubiquitin chains [13,24,32]. How JAMM/MPN${}^{+}$ DUBs cleave ubiquitin chains with K63-linkage specificity has been explained by the crystal structure of AMSH-LP bound to K63-linked di-ubiquitin: Two insertion loops orient the distal and proximal ubiquitin in order to place the isopeptide bond at lysine 63 in direct proximity to the active site [80]. While insertion 2 of AMSH-LP does not have a structural counterpart in BRCC36, insertion 1 contacts both the distal and proximal ubiquitin, explaining the observed linkage specificity [24].

### 5.3. Length Selectivity

Fully assembled BRCA1-A and BRISC complexes show substantial acceleration of degradation when ubiquitin chains are four or more ubiquitin moieties long [25]. Length selectivity is most likely conferred by ubiquitin binding domains on BRE and, possibly, MERIT40 [25,40].

### 5.4. Mixed Chains

RAP80 binds mixed SUMO-K63-linked ubiquitin chains with high affinity [47,48]. BRCA1-A complex preferentially degrades mixed SUMO-ubiquitin chains and this activity depends on RAP80, which selectively increases the local concentration of mixed chains [25].

### 5.5. SHMT2 Inhibits BRISC

Control of IFNAR1 and HIV-1 Tat protein degradation by BRISC relies on the presence of SHMT2 [67,68]. SHMT2, the serine hydroxy methyltransferase, is a metabolic enzyme (504 aa, 56 kDa) that converts serine to glycine and a tetrahydrofolate-bound one carbon unit [81]. SHMT2 is expressed in two isoforms: the long, mitochondrial form which carries an N-terminal mitochondrial import signal and is imported into mitochondria, and the isoform SHMT2α (483 aa, 54 kDa), which lacks the mitochondrial import signal and localizes to the cytosol and nucleus [82]. SHMT2 requires cofactor PLP for enzymatic activity. While apo-SHMT2 forms dimers in an open conformation and with an unstructured active site region, upon binding of PLP the active site becomes structured, the angle between the subunits changes to a closed conformation, and the dimers dimerize to form tetrameric, enzymatically active SHMT2 [81].

**Figure 4:**
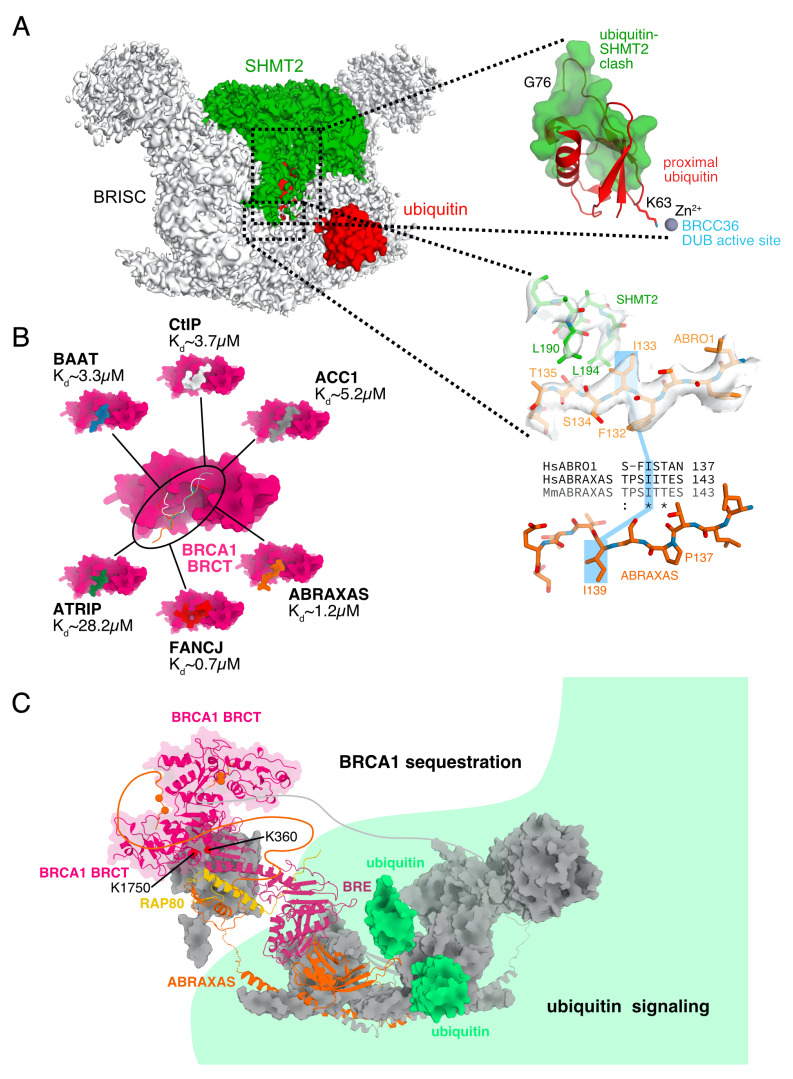
(**A**) Inactivation of BRISC by SHMT2α binding. A cryo-EM map of BRISC-SHMT2α complex is shown with a di-ubiquitin fit into the active site using the structure of AMSH-LP with bound di-ubiquitin [80]. The clash between SHMT2 and the proximal ubiquitin is shown in a zoom-out. BRCA1-A complex does not bind SHMT2, because residue I133 of Abro1, which is essential for the interaction, is conserved, but flipped by 180${}^{\circ }$ due to a proline insertion at position P137. The cryo-EM map of BRISC-SHMT2α is shown in white while the side chains of SHMT2 and ABRO1 are shown in green and light orange, respectively. The structure of ABRAXAS that corresponds to the part of ABRO1 shown is depicted below in orange (BRCA1-A and BRISC-SHMT2α structure: [25]). (**B**) ACC1, ATRIP, BAAT, CtIP, and FANCJ compete with the phosphorylated C-terminus of ABRAXAS for the same binding site on BRCA1. Binding affinities measured for the phosphorylated peptide only are in the micromolar range. The entire BRCA1-A complex however binds with nanomolar affinity (ABRAXAS-BRCA1 complex structure: [30], ACC1-BRCA1 complex structure: [83], ATRIP-BRCA1 complex structure: [84], BAAT-BRCA1 complex structure: [84], CtIP-BRCA1 complex structure: [85], FANCJ-BRCA1 complex structure: [86]). (**C**) Crosslinking data suggests that the BRCA1 BRCT domain is localized at the edge of the arc, distal from the active sites of the complex. This model explains why sequestration of BRCA1 by BRCA1-A does not affect the DUB activity of the complex. BRCA1-A can be considered to be divided into a ubiquitin signaling zone (green) and a BRCA1 sequestration zone that are functionally independent (BRCA1-A structure: [25], AMSH-LP-substrate complex structure used for modelling ubiquitin at the active site of BRCA1-A: [80], structure of BRCA1 BRCT dimer with bound ABRAXAS C-terminal phosphorylated peptide: [30]).

Purified BRISC and apo-SHMT2α spontaneously form a complex with low nanomolar affinity upon mixing [25,26]. In the BRISC-SHMT2 complex, two subunits of SHMT2α are bound in the center of the BRISC arc, directly on top of the JAMM/MPN superdimer of subunits ABRO1 and BRCC36 [25,26] (Figure 1A and Figure 4A). The main interaction between BRISC and SHMT2α is mediated by ABRO1, which binds two hydrophobic residues, L190 and L194, of SHMT2α, but SHMT2α contacts BRCC36 and BRE as well [25,26].

SHMT2 binds BRISC in the open conformation with the active site of SHMT2 unstructured [25,26]. As BRISC binds the interaction surface that mediates the tetramerization of PLP-bound SHMT2, SHMT2 tetramerization and BRISC binding are mutually exclusive, explaining why BRISC-bound SHMT2 shows no enzymatic activity [25,26,68]. PLP-bound SHMT2 shows lower affinity for BRISC, which can be further reduced by supplementation of free PLP [26].

Due to the position of SHMT2 inside the BRISC arc, proximal to the active site of BRCC36, bound SHMT2 sterically excludes ubiquitin chains from the active site of BRISC and renders the complex inactive as a DUB [25,26]. The DUB activity of BRISC can be controlled by addition of PLP, which drives the equilibrium from BRISC-bound apo-SHMT2 to PLP-bound SHMT2 tetramer and free, active BRISC [26].

The region of ABRAXAS corresponding to the SHMT2α binding region of ABRO1 is conserved, but the insertion of a proline at position P144 flips the position of the side chain of the isoleucin corresponding to I133 in ABRO1 away from the surface, obviating hydrophobic interaction with L194 of SHMT2α [25] (Figure 4A). As a result, despite being otherwise structurally very similar, BRCA1-A does not bind SHMT2α, which is present in the nucleus [25,82].

## 6. High-Affinity Binding of BRCA1

### 6.1. ABRAXAS-BRCA1 Complex Formation

BRCA1 sequestration by BRCA1-A complex is essential to successful DNA repair regulation. How does BRCA1-A bind BRCA1 with affinity high enough to withdraw it from the site of damage? Two domains located near the C-terminus of BRCA1, BRCT1 (residues 1642–1736) and BRCT2 (residues 1756–1855), together form the BRCT domain, an important hub in BRCA1 signaling [87]. BRCA1 BRCT is a phosphopeptide binding domain that mediates complex formation with phosphorylated proteins [88]. The C-terminus of ABRAXAS contains two phosphorylation sites: S406 is constitutively phosphorylated and DNA damage induces phosphorylation of S404 [18,30]. The C-terminal carboxyl group, F409, and phosphorylated S406 of ABRAXAS are specifically recognized by BRCA1 BRCT and mediate binding [30]. Additional phosphorylation of ABRAXAS S404 enables a salt bridge to a second BRCA1 BRCT molecule and induces dimerization [30].

The BRCA1 BRCT domain binds several other phosphorylated proteins besides ABRAXAS, including ACC1, ATRIP, BAAT, BACH1, and CtIP [83,84,85,86,89]. Binding affinities of synthetic phosphopeptides for the BRCA1 BRCT domain have been determined by isothermal titration calorimetry and range from K${}_{d}$∼ 0.7 μM (BACH1) to K${}_{d}$∼ 28.2 μM (ATRIP), with ABRAXAS found among the tighter binders at K${}_{d}$∼ 1.2 μM [84,85,90]. Since BRCA1 BRCT contains only one phosphopeptide binding site, complexes with BRCA1 form in a mutually exclusive manner and ABRAXAS must compete with resection promoting complexes for the same binding site (Figure 4B). If the binding affinities of phosphorylated peptides to BRCA1 BRCT all range in the low micromolar range, what enables ABRAXAS to withdraw BRCA1 from the site of damage and sequester it away from resection promoting complexes? This discrepancy was resolved when the affinity of BRCA1 BRCT for the entire BRCA1-A complex with phosphorylated ABRAXAS was measured. Instead of an affinity of K${}_{d}$∼ 1.2 μM measured for the phosphopeptide, ABRAXAS integrated into the entire BRCA1-A complex binds BRCA1 BRCT with nanomolar affinity at K${}_{d}$∼ 80 nM [25]. Remarkably, the affinity of BRCA1-A complex for BRCA1 BRCT not only exceeds the affinity measured for all phosphopeptides derived from natural proteins, but also surpasses the affinity measured for a phosphopeptide that had been in vitro evolved in order to maximize binding strength (K${}_{d}$∼ 400 nM) [91].

A combination of several factors is thought to cause the exceptional affinity between BRCA1-A and BRCA1: BRCA1-A forms a single arc, which consists of a dimer of hetero-pentamers (ABRAXAS, BRCC36, BRE, MERIT40, and RAP80) [25]. The two tethered ABRAXAS C-termini function as bivalent ligands, doubling the association rate constant [92]. In addition, the BRCA1 BRCT dimerization effected by ABRAXAS pSer404/406 leads to forced proximity that additionally decreases the dissociation constant [92]. Together, these structural properties of BRCA1-A and BRCA1 BRCT qualitatively explain the observed increase of avidity when compared to the phosphopeptide. In addition, there is evidence that BRCA1 BRCT interacts with other subunits of BRCA1-A besides the ABRAXAS C-terminus, such as BRE or MERIT40 [25,45]. Interaction with MERIT40 and BRE would place BRCA1 BRCT at the periphery of the BRCA1-A arc, distal from the active DUB site. Functional independence of DUB function from BRCA1 sequestration was indeed confirmed: BRCA1 BRCT binding to BRCA1-A did not reduce the DUB activity against RNF4-generated long mixed SUMO-ubiquitin chains [25]. This means that BRCA1-A remains a fully active DUB while sequestering BRCA1 in DNA repair *foci*, actively shaping their extent in the process (Figure 4C).

### 6.2. BRCA1 Complexes

BRCA1 binding to protein complexes has profound biological impact, from regulation of fatty acid synthesis to the promotion of DNA resection [93,94]. Due to sterical constraints, binding of phosphorylated proteins ABRAXAS, ACC1, ATRIP, BAAT, BACH1, or CtIP to the BRCT domain of BRCA1 is mutually exclusive [95] (Figure 4B). With DNA damage induced or cell-cycle controlled phosphorylation of the BRCA1 binding sites controlling the association of BRCA1 complexes, there likely is competition between them for BRCA1. The nanomolar affinity measured between BRCA1-A and BRCA1 cannot be compared to the substantially lower affinities that were measured for interactions with the other proteins when the only data available for them are affinity measurements employing peptides rather than entire assembled complexes [25,90]. Precise measurements of the binding affinity of BRCA1 to fully assembled DNA-repair complexes and investigation of their competition for BRCA1 will advance our understanding of the DNA repair process.

## 7. BRCA1-A and BRISC in Human Health

With important roles in DNA repair, immune regulation, mitosis, and hematopoiesis, BRCA1-A and BRISC substantially impact human health. Targeted disruption experiments found neither complex to be essential for survival or reproduction in mice, but implicated BRCA1-A in cancer suppression [68,96,97,98,99]. Point mutations that disrupt BRCA1 sequestration by BRCA1-A have been identified in human cancer patients: A mutation in the nuclear localization sequence of ABRAXAS (R361Q) results in a completely assembled, functional BRCA1-A complex that is excluded from the nucleus [36]. A deletion in the linker between the UIM domains of RAP80 (ΔE81) found in cancer patients disrupts ubiquitin binding, preventing BRCA1 sequestration to DNA repair *foci* [46,100,101]. A missense mutation of the C-terminal residue of ABRAXAS (F409C), which was discovered in an endometrial tumor, removes the terminal phenylalanine essential for high-affinity binding to BRCA1 [30,96].

BRCA1-A mutations that disrupt BRCA1 sequestration have been identified in breast cancer patients, suggesting a tissue tropism similar to BRCA1 mutations [36,101]. BRCA1 mutations are always heterozygous, because BRCA1 is essential for embryonic development [102]. Loss of heterozygosity eliminates the wild-type allele in proliferative female tissues, which preferentially use the homologous recombination pathway, giving rise to the striking tissue tropism seen in BRCA1 breast cancer [103]. Although BRCA1-A complex is not essential for embryonic development [104], all subunits of BRCA1-A with the exception of BRCC36 are located on autosomes and mutations are likely to be heterozygous. From a biochemical perspective, the two best-characterized mutations found in breast cancer, if heterozygous, would not be expected to disrupt the working, wild-type BRCA1-A, similar to many known BRCA1 mutations [36,101,102]. Loss of heterozygosity in breast tissue would disrupt BRCA1-A function and cause genomic instability. As cancer-associated mutations in BRCA1-A subunits have been identified in a wide variety of tumors, further research is needed to fully characterize each mutation and assess the cancer risk [105].

Expression levels of BRCA1-A subunits influence carcinogenesis: Breast cancer cells show reduced expression of RAP80 [106], while overexpression of MERIT40 has been found in epithelial ovarian cancer [107]. Any mutation disrupting BRCA1-A assembly, targeting, enzymatic function, or BRCA1-A-BRCA1 complex formation is expected to increases cancer risk. Structural data will facilitate the functional evaluation of cancer-associated somatic mutations, which have been identified in all subunits of BRCA1-A [105].

The response to cancer treatments is affected by the DNA repair capabilities of the tumor cells and expression level of BRCA1-A subunits can be used to anticipate therapy outcome. Higher expression of BRE predicts a favourable treatment outcome in acute myeloid leukemia and breast cancer [108,109,110,111].

Human patients with a congenital deletion of BRCC36—expected to disrupt the assembly and function of both BRCA1-A and BRISC complex—are affected by severe symptoms including moyamoya angiopathy, short stature, facial dysmorphism and infertility [104]. Symptoms such as cataracts and early hair graying point to an impairment of DNA repair processes in line with a disrupted BRCA1-A complex. The mechanism behind the reported developmental defects, angiopathy, and infertility, however, is currently not fully understood and points to additional functions of BRCA1-A and BRISC in human physiology.

The discovery of interferon response regulation by BRISC promises a new approach to the treatment of human immune disorders, especially if BRISC DUB inhibtion by SHMT2 can be regulated by small molecules such as PLP in a clinical setting [26,68]. As tumor-specific T-cells infiltrate the hypoxic areas inside tumors, they lose the IFNAR1 receptors essential for their function [112]. SHMT2 is a hypoxia-inducible factor and frequently overexpressed in tumor cells, at concentrations that could inactivate BRISC and affect IFNAR1 [25,113,114]. Further research is needed to elucidate how SHMT2, BRISC, and IFNAR1 interact in health and disease.

## 8. Summary and Outlook

Nearly two decades of research have revealed BRCA1-A and BRISC to be sophisticated molecular machines for ubiquitin signaling. Sharing the same core and enzymatic subunit, their physiological functions are strikingly distinct.

Understanding the function of BRCA1-A in DNA repair promises better treatment of cancer and congenital diseases. Pharmacological compounds that disrupt BRCA1 complex formation are developed for use in cancer therapy: Chemosensitivity of cancer cells in response to cisplatin therapy is attenuated by DNA repair. Bractoppin is an inhibitor for phosphopeptide binding to BRCA1 BRCT that prevents association with BACH1 and ABRAXAS phosphopeptide in vitro, and sensitizes cells to radiation damage [115,116].

Disruption of BRCA1 sequestration by BRCA1-A hyperactivates HR. Most mammalian cells repair DNA damage with the NHEJ pathway, fundamentally limiting the use of recombination during gene therapy by CRISPR/Cas9 [117]. Up-regulation of HR by specific disruption of BRCA1-A mediated BRCA1 sequestration could supplement methods that rely on chemical inhibition of DNA repair proteins, cell cycle arrest, or sophisticated genetic approaches [118] to enable CRISPR/Cas9-based therapies for human congenital diseases.

The discovery that BRISC regulates the interferon response promises the development of pharmacological compounds to attenuate inflammation. IFNAR1 is an essential mediator of the human interferon response and a promising drug target [119]. The study of BRISC-SHMT2 has revealed how the abundance of IFNAR1 on the cell surface is controlled. The exciting discovery that association with SHMT2 is controlled by PLP promises pharmaceutical compounds able to modify the abundance of IFNAR1, allowing new therapies for cancer and autoimmune disorders.

In addition to the aforementioned medical and biotechnological promises, understanding BRCA1-A and BRISC also opens the gate to a new appraisal of the capabilities of DUBs in general. When BRCC36 is observed in isolation, e.g., in an in vitro high-throughput assessment of recombinantly expressed protein, it appears to be an unregulated DUB with strict linkage specificity and average activity [13]. Yet, in vivo BRCC36 is capable of performing two distinct, essential biological functions with high precision. Activity, specificity, targeting, and regulation of BRCC36 differ starkly in BRCA1-A and BRISC, and these specialized functions become only apparent in context of the assembled molecular machines. BRCC36 is one of just ∼100 DUBs found in human cells that counter the activity of ∼600 highly specific and tightly regulated E3 ligases that ubiquitinate ∼20,000 individual sites on the human proteome [12]. BRCC36 is the first example of an active DUB that is functionalized for different purposes by embedding into different molecular machines, where other subunits convey targeting, specificity and regulation. This theme could have more general validity beyond BRCC36: Many human DUBs are incorporated into larger assemblies [120]. Functionalization of the same DUB for different tasks by incorporation into different complexes would allow countering the complexities of ubiquitin E3 ligation with a limited repertoire of DUBs.

## Figures and Tables

**Table 1:** Subunits of BRCA1-A and BRISC complex, and binding partners.

Name	Gene Names	Size (aa)	Size (kDa)	Domains	Associated with	Constitutive
ABRAXAS	ABRAXAS1, ABRA1, CCDC98, FAM175A, UNQ496/PRO1013	409	36.7	JAMM/MPN${}^{-}$, BRCA1 binding	BRCA1-A	subunit
ABRO1	ABRAXAS2, FAM175B, KIAA0157	415	46.9	JAMM/MPN${}^{-}$, SHMT2α and LNK bdg.	BRISC	subunit
BRCC36	BRCC3, C6.1A, CXorf53	316	36.0	JAMM/MPN${}^{+}$	BRCA1-A, BRISC	subunit
BRE	BABAM2, BRCC45	383	43.6	UEV	BRCA1-A, BRISC	subunit
MERIT40	BABAM1, C19orf62, NBA1, HSPC142	329	36.6	VWA	BRCA1-A, BRISC	subunit
RAP80	UIMC1, RXRIP110	719	79.7	SIM, twin UIM, AIR	BRCA1-A	subunit
BRCA1	RNF53	1863	207.7	RING, BRCT	BRCA1-A	regulated binding
SHMT2α	GLYM	483	53.5	SHMT	BRISC	regulated binding

## Abbreviations

DUB	Deubiquitinating enzyme
HR	Homologous Recombination
JAMM/MPN${}^{+}$	JAB1/MPN/MOV34 metalloenzyme domain
NHEJ	Non-Homologous End Joining
PLP	pyridoxal 5’ -phosphate
RWD	RING finger and WD repeat containing proteins and DEXDc helicases
SIM	SUMO-Interacting Motif
UEV	Ubiquitin E2 Variant domain
UIM	Ubiquitin-Interacting Motif
VWA	Von Willebrand factor (vWF) type A domain

## References

[1] Komander D., Rape M. (2012). The Ubiquitin Code. Annu. Rev. Biochem..

[2] Kauko A., Lehto K. (2018). Eukaryote specific folds: Part of the whole. Proteins Struct. Funct. Bioinform..

[3] Delley C.L., Müller A.U., Ziemski M., Weber-Ban E. (2017). Prokaryotic Ubiquitin-Like Protein and Its Ligase/Deligase Enyzmes. J. Mol. Biol..

[4] Polge C., Uttenweiler-Joseph S., Leulmi R., Heng A.E., Burlet-Schiltz O., Attaix D., Taillandier D. (2013). Deciphering the ubiquitin proteome: Limits and advantages of high throughput global affinity purification-mass spectrometry approaches. Int. J. Biochem. Cell Biol..

[5] Hospenthal M.K., Freund S.M.V., Komander D. (2013). Assembly, analysis and architecture of atypical ubiquitin chains. Nat. Struct. Mol. Biol..

[6] Ye Y., Blaser G., Horrocks M.H., Ruedas-Rama M.J., Ibrahim S., Zhukov A.A., Orte A., Klenerman D., Jackson S.E., Komander D. (2012). Ubiquitin chain conformation regulates recognition and activity of interacting proteins. Nature.

[7] Bremm A., Freund S.M.V., Komander D. (2010). Lys11-linked ubiquitin chains adopt compact conformations and are preferentially hydrolyzed by the deubiquitinase Cezanne. Nat. Struct. Mol. Biol..

[8] Komander D., Reyes-Turcu F., Licchesi J.D.F., Odenwaelder P., Wilkinson K.D., Barford D. (2009). Molecular discrimination of structurally equivalent Lys 63-linked and linear polyubiquitin chains. EMBO Rep..

[9] Kwon Y.T., Ciechanover A. (2017). The Ubiquitin Code in the Ubiquitin-Proteasome System and Autophagy. Trends Biochem. Sci..

[10] Erpapazoglou Z., Walker O., Haguenauer-Tsapis R. (2014). Versatile Roles of K63-Linked Ubiquitin Chains in Trafficking. Cells.

[11] Clague M.J., Urbé S. (2006). Endocytosis: The DUB version. Trends Cell Biol..

[12] Leznicki P., Kulathu Y. (2017). Mechanisms of regulation and diversification of deubiquitylating enzyme function. J. Cell Sci..

[13] Ritorto M.S., Ewan R., Perez-Oliva A.B., Knebel A., Buhrlage S.J., Wightman M., Kelly S.M., Wood N.T., Virdee S., Gray N.S. (2014). Screening of DUB activity and specificity by MALDI-TOF mass spectrometry. Nat. Commun..

[14] Shin J.Y., Muniyappan S., Tran N.N., Park H., Lee S.B., Lee B.H. (2020). Deubiquitination Reactions on the Proteasome for Proteasome Versatility. Int. J. Mol. Sci..

[15] Dong Y., Hakimi M.A., Chen X., Kumaraswamy E., Cooch N.S., Godwin A.K., Shiekhattar R. (2003). Regulation of BRCC, a holoenzyme complex containing BRCA1 and BRCA2, by a signalosome-like subunit and its role in DNA repair. Mol. Cell.

[16] Kim H., Chen J., Yu X. (2007). Ubiquitin-Binding Protein RAP80 Mediates BRCA1-Dependent DNA Damage Response. Science.

[17] Sobhian B., Shao G., Lilli D.R., Culhane A.C., Moreau L.A., Xia B., Livingston D.M., Greenberg R.A. (2007). RAP80 Targets BRCA1 to Specific Ubiquitin Structures at DNA Damage Sites. Science.

[18] Wang B., Matsuoka S., Ballif B.A., Zhang D., Smogorzewska A., Gygi S.P., Elledge S.J. (2007). Abraxas and RAP80 Form a BRCA1 Protein Complex Required for the DNA Damage Response. Science.

[19] Cooper E.M., Cutcliffe C., Kristiansen T.Z., Pandey A., Pickart C.M., Cohen R.E. (2009). K63-specific deubiquitination by two JAMM/MPN+ complexes: BRISC-associated Brcc36 and proteasomal Poh1. EMBO J..

[20] Feng L., Huang J., Chen J. (2009). MERIT40 facilitates BRCA1 localization and DNA damage repair. Genes Dev..

[21] Shao G., Patterson-Fortin J., Messick T.E., Feng D., Shanbhag N., Wang Y., Greenberg R.A. (2009). MERIT40 controls BRCA1-Rap80 complex integrity and recruitment to DNA double-strand breaks. Genes Dev..

[22] Wang B., Hurov K., Hofmann K., Elledge S.J. (2009). NBA1, a new player in the Brca1 A complex, is required for DNA damage resistance and checkpoint control. Genes Dev..

[23] Hu X., Kim J.A., Castillo A., Huang M., Liu J., Wang B. (2011). NBA1/MERIT40 and BRE Interaction Is Required for the Integrity of Two Distinct Deubiquitinating Enzyme BRCC36-containing Complexes. J. Biol. Chem..

[24] Zeqiraj E., Tian L., Piggott C.A., Pillon M.C., Duffy N.M., Ceccarelli D.F., Keszei A.F.A., Lorenzen K., Kurinov I., Orlicky S. (2015). Higher-Order Assembly of BRCC36–KIAA0157 Is Required for DUB Activity and Biological Function. Mol. Cell.

[25] Rabl J., Bunker R.D., Schenk A.D., Cavadini S., Gill M.E., Abdulrahman W., Andrés-Pons A., Luijsterburg M.S., Ibrahim A.F.M., Branigan E. (2019). Structural Basis of BRCC36 Function in DNA Repair and Immune Regulation. Mol. Cell.

[26] Walden M., Tian L., Ross R.L., Sykora U.M., Byrne D.P., Hesketh E.L., Masandi S.K., Cassel J., George R., Ault J.R. (2019). Metabolic control of BRISC–SHMT2 assembly regulates immune signalling. Nature.

[27] Block-Schmidt A.S., Dukowic-Schulze S., Wanieck K., Reidt W., Puchta H. (2011). BRCC36A is epistatic to BRCA1 in DNA crosslink repair and homologous recombination in Arabidopsis thaliana. Nucleic Acids Res..

[28] Uhlén M., Zhang C., Lee S., Sjöstedt E., Fagerberg L., Bidkhori G., Benfeitas R., Arif M., Liu Z., Edfors F. (2017). A pathology atlas of the human cancer transcriptome. Science.

[29] Uhlén M., Fagerberg L., Hallström B.M., Lindskog C., Oksvold P., Mardinoglu A., Sivertsson Å., Kampf C., Sjöstedt E., Asplund A. (2015). Tissue-based map of the human proteome. Science.

[30] Wu Q., Paul A., Su D., Mehmood S., Foo T.K., Ochi T., Bunting E.L., Xia B., Robinson C.V., Wang B. (2016). Structure of BRCA1-BRCT/Abraxas Complex Reveals Phosphorylation-Dependent BRCT Dimerization at DNA Damage Sites. Mol. Cell.

[31] Wang B., Elledge S.J. (2007). Ubc13/Rnf8 ubiquitin ligases control foci formation of the Rap80/Abraxas/Brca1/Brcc36 complex in response to DNA damage. Proc. Natl. Acad. Sci. USA.

[32] Cooper E.M., Boeke J.D., Cohen R.E. (2010). Specificity of the BRISC deubiquitinating enzyme is not due to selective binding to Lys63-linked polyubiquitin. J. Biol. Chem..

[33] Mevissen T.E.T., Komander D. (2017). Mechanisms of Deubiquitinase Specificity and Regulation. Annu. Rev. Biochem..

[34] Lingaraju G.M., Bunker R.D., Cavadini S., Hess D., Hassiepen U., Renatus M., Fischer E.S., Thomä N.H. (2014). Crystal structure of the human COP9 signalosome. Nature.

[35] Pathare G.R., Nagy I., Śledź P., Anderson D.J., Zhou H.J., Pardon E., Steyaert J., Förster F., Bracher A., Baumeister W. (2014). Crystal structure of the proteasomal deubiquitylation module Rpn8-Rpn11. Proc. Natl. Acad. Sci. USA.

[36] Solyom S., Aressy B., Pylkäs K., Patterson-Fortin J., Hartikainen J.M., Kallioniemi A., Kauppila S., Nikkilä J., Kosma V.M., Mannermaa A. (2012). Breast cancer-associated Abraxas mutation disrupts nuclear localization and DNA damage response functions. Sci. Transl. Med..

[37] Donaghy R., Han X., Rozenova K., Lv K., Jiang Q., Doepner M., Greenberg R.A., Tong W. (2019). The BRISC deubiquitinating enzyme complex limits hematopoietic stem cell expansion by regulating JAK2 K63-ubiquitination. Blood.

[38] Hurley J.H., Lee S., Prag G. (2006). Ubiquitin-binding domains. Biochem. J..

[39] Hicke L., Schubert H.L., Hill C.P. (2005). Ubiquitin-binding domains. Nat. Rev. Mol. Cell Biol..

[40] Kyrieleis O.J.P., McIntosh P.B., Webb S.R., Calder L.J., Lloyd J., Patel N.A., Martin S.R., Robinson C.V., Rosenthal P.B., Smerdon S.J. (2016). Three-Dimensional Architecture of the Human BRCA1-A Histone Deubiquitinase Core Complex. Cell Rep..

[41] Anamika, Spyracopoulos L. (2016). Molecular Basis for Phosphorylation-dependent SUMO Recognition by the DNA Repair Protein RAP80. J. Biol. Chem..

[42] Sato Y., Yoshikawa A., Mimura H., Yamashita M., Yamagata A., Fukai S. (2009). Structural basis for specific recognition of Lys 63-linked polyubiquitin chains by tandem UIMs of RAP80. EMBO J..

[43] Kang Y., Chen X., Lary J.W., Cole J.L., Walters K.J. (2007). Defining how Ubiquitin Receptors hHR23a and S5a Bind Polyubiquitin. J. Mol. Biol..

[44] Guettler S., LaRose J., Petsalaki E., Gish G., Scotter A., Pawson T., Rottapel R., Sicheri F. (2011). Structural Basis and Sequence Rulesfor Substrate Recognition by Tankyrase Explain the Basis for Cherubism Disease. Cell.

[45] Vikrant, Sawant U.U., Varma A.K. (2014). Role of MERIT40 in stabilization of BRCA1 complex: A protein-protein interaction study. Biochem. Biophys. Res. Commun..

[46] Anamika, Markin C.J., Rout M.K., Spyracopoulos L. (2014). Molecular basis for impaired DNA damage response function associated with the RAP80 ΔE81 defect. J. Biol. Chem..

[47] Guzzo C.M., Berndsen C.E., Zhu J., Gupta V., Datta A., Greenberg R.A., Wolberger C., Matunis M.J. (2012). RNF4-dependent hybrid SUMO-ubiquitin chains are signals for RAP80 and thereby mediate the recruitment of BRCA1 to sites of DNA damage. Sci. Signal..

[48] Hu X., Paul A., Wang B. (2012). Rap80 Protein Recruitment to DNA Double-strand Breaks Requires Binding to Both Small Ubiquitin-like Modifier (SUMO) and Ubiquitin Conjugates. J. Biol. Chem..

[49] Bian C., Wu R., Cho K., Yu X. (2012). Loss of BRCA1-A complex function in RAP80 null tumor cells. PLoS ONE.

[50] Patterson-Fortin J., Shao G., Bretscher H., Messick T.E., Greenberg R.A. (2010). Differential Regulation of JAMM Domain Deubiquitinating Enzyme Activity within the RAP80 Complex. J. Biol. Chem..

[51] Symington L.S., Gautier J. (2011). Double-Strand Break End Resection and Repair Pathway Choice. Annu. Rev. Genet..

[52] Aparicio T., Baer R., Gautier J. (2014). DNA double-strand break repair pathway choice and cancer. DNA Repair.

[53] Dantuma N.P., Pfeiffer A. (2016). Real Estate in the DNA Damage Response: Ubiquitin and SUMO Ligases Home in on DNA Double-Strand Breaks. Front. Genet..

[54] Nie M., Boddy M. (2016). Cooperativity of the SUMO and Ubiquitin Pathways in Genome Stability. Biomolecules.

[55] Rogakou E.P., Boon C., Redon C., Bonner W.M. (1999). Megabase chromatin domains involved in DNA double-strand breaks in vivo. J. Cell Biol..

[56] Kakarougkas A., Jeggo P.A. (2014). DNA DSB repair pathway choice: An orchestrated handover mechanism. Br. J. Radiol..

[57] Smeenk G., Mailand N. (2016). Writers, Readers, and Erasers of Histone Ubiquitylation in DNA Double-Strand Break Repair. Front. Genet..

[58] Thorslund T., Ripplinger A., Hoffmann S., Wild T., Uckelmann M., Villumsen B., Narita T., Sixma T.K., Choudhary C., Bekker-Jensen S. (2015). Histone H1 couples initiation and amplification of ubiquitin signalling after DNA damage. Nature.

[59] Mattiroli F., Vissers J.H.A., van Dijk W.J., Ikpa P., Citterio E., Vermeulen W., Marteijn J.A., Sixma T.K. (2012). RNF168 ubiquitinates K13-15 on H2A/H2AX to drive DNA damage signaling. Cell.

[60] Smeenk G., van Attikum H. (2013). The Chromatin Response to DNA Breaks: Leaving a Mark on Genome Integrity. Annu. Rev. Biochem..

[61] Galanty Y., Belotserkovskaya R., Coates J., Jackson S.P. (2012). RNF4, a SUMO-targeted ubiquitin E3 ligase, promotes DNA double-strand break repair. Genes Dev..

[62] Kakarougkas A., Ismail A., Katsuki Y., Freire R., Shibata A., Jeggo P.A. (2013). Co-operation of BRCA1 and POH1 relieves the barriers posed by 53BP1 and RAP80 to resection. Nucleic Acids Res..

[63] Goldstein M., Kastan M.B. (2015). Repair versus Checkpoint Functions of BRCA1 Are Differentially Regulated by Site of Chromatin Binding. Cancer Res..

[64] Hu Y., Scully R., Sobhian B., Xie A., Shestakova E., Livingston D.M. (2011). RAP80-directed tuning of BRCA1 homologous recombination function at ionizing radiation-induced nuclear foci. Genes Dev..

[65] Shao G., Lilli D.R., Patterson-Fortin J., Coleman K.A., Morrissey D.E., Greenberg R.A. (2009). The Rap80-BRCC36 de-ubiquitinating enzyme complex antagonizes RNF8-Ubc13-dependent ubiquitination events at DNA double strand breaks. Proc. Natl. Acad. Sci. USA.

[66] Ng H.M., Wei L., Lan L., Huen M.S.Y. (2016). The Lys 63-deubiquitylating Enzyme BRCC36 Limits DNA Break Processing and Repair. J. Biol. Chem..

[67] Xu M., Moresco J.J., Chang M., Mukim A., Smith D., Diedrich J.K., Yates J.R., Jones K.A. (2018). SHMT2 and the BRCC36/BRISC deubiquitinase regulate HIV-1 Tat K63-ubiquitylation and destruction by autophagy. PLoS Pathog..

[68] Zheng H., Gupta V., Patterson-Fortin J., Bhattacharya S., Katlinski K., Wu J., Varghese B., Carbone C.J., Aressy B., Fuchs S.Y. (2013). A BRISC-SHMT complex deubiquitinates IFNAR1 and regulates interferon responses. Cell Rep..

[69] Schoggins J.W. (2019). Interferon-Stimulated Genes: What Do They All Do?. Annu. Rev. Virol..

[70] Bhattacharya S., Katlinski K.V., Reichert M., Takano S., Brice A., Zhao B., Yu Q., Zheng H., Carbone C.J., Katlinskaya Y.V. (2014). Triggering ubiquitination of IFNAR1 protects tissues from inflammatory injury. EMBO Mol. Med..

[71] de Weerd N.A., Vivian J.P., Nguyen T.K., Mangan N.E., Gould J.A., Braniff S.J., Zaker-Tabrizi L., Fung K.Y., Forster S.C., Beddoe T. (2013). Structural basis of a unique interferon-*β* signaling axis mediated via the receptor IFNAR1. Nat. Immunol..

[72] Liu J., Carvalho L.P., Bhattacharya S., Carbone C.J., Kumar K.G.S., Leu N.A., Yau P.M., Donald R.G.K., Weiss M.J., Baker D.P. (2009). Mammalian Casein Kinase 1α and Its Leishmanial Ortholog Regulate Stability of IFNAR1 and Type I Interferon Signaling. Mol. Cell. Biol..

[73] Kumar K.G.S., Krolewski J.J., Fuchs S.Y. (2004). Phosphorylation and Specific Ubiquitin Acceptor Sites are Required for Ubiquitination and Degradation of the IFNAR1 Subunit of Type I Interferon Receptor. J. Biol. Chem..

[74] Kumar K.G.S., Barriere H., Carbone C.J., Liu J., Swaminathan G., Xu P., Li Y., Baker D.P., Peng J., Lukacs G.L. (2007). Site-specific ubiquitination exposes a linear motif to promote interferon-*α* receptor endocytosis. J. Cell Biol..

[75] Lata S., Mishra R., Banerjea A.C. (2018). Proteasomal Degradation Machinery: Favorite Target of HIV-1 Proteins. Front. Microbiol..

[76] Yan K., Li L., Wang X., Hong R., Zhang Y., Yang H., Lin M., Zhang S., He Q., Zheng D. (2015). The deubiquitinating enzyme complex BRISC is required for proper mitotic spindle assembly in mammalian cells. J. Cell Biol..

[77] Tripathi E., Smith S. (2017). Cell cycle-regulated ubiquitination of tankyrase 1 by RNF8 and ABRO1/BRCC36 controls the timing of sister telomere resolution. EMBO J..

[78] Feng L., Wang J., Chen J. (2010). The Lys63-specific Deubiquitinating Enzyme BRCC36 Is Regulated by Two Scaffold Proteins Localizing in Different Subcellular Compartments. J. Biol. Chem..

[79] Worden E.J., Padovani C., Martin A. (2014). Structure of the Rpn11-Rpn8 dimer reveals mechanisms of substrate deubiquitination during proteasomal degradation. Nat. Struct. Mol. Biol..

[80] Sato Y., Yoshikawa A., Yamagata A., Mimura H., Yamashita M., Ookata K., Nureki O., Iwai K., Komada M., Fukai S. (2008). Structural basis for specific cleavage of Lys63-linked polyubiquitin chains. Nature.

[81] Giardina G., Brunotti P., Fiascarelli A., Cicalini A., Costa M.G.S., Buckle A.M., di Salvo M.L., Giorgi A., Marani M., Paone A. (2015). How pyridoxal 5’-phosphate differentially regulates human cytosolic and mitochondrial serine hydroxymethyltransferase oligomeric state. FEBS J..

[82] Anderson D.D., Stover P.J. (2009). SHMT1 and SHMT2 Are Functionally Redundant in Nuclear De novo Thymidylate Biosynthesis. PLoS ONE.

[83] Shen Y., Tong L. (2008). Structural Evidence for Direct Interactions between the BRCT Domains of Human BRCA1 and a Phospho-peptide from Human ACC1. Biochemistry.

[84] Liu X., Ladias J.A.A. (2013). Structural Basis for the BRCA1 BRCT Interaction with the Proteins ATRIP and BAAT1. Biochemistry.

[85] Varma A.K., Brown R.S., Birrane G., Ladias J.A.A. (2005). Structural basis for cell cycle checkpoint control by the BRCA1-CtIP complex. Biochemistry.

[86] Shiozaki E.N., Gu L., Yan N., Shi Y. (2004). Structure of the BRCT repeats of BRCA1 bound to a BACH1 phosphopeptide: Implications for signaling. Mol. Cell.

[87] Huyton T., Bates P.A., Zhang X., Sternberg M.J., Freemont P.S. (2000). The BRCA1 C-terminal domain: Structure and function. Mutat. Res..

[88] Yu X., Chini C.C.S., He M., Mer G., Chen J. (2003). The BRCT domain is a phospho-protein binding domain. Science.

[89] Clapperton J.A., Manke I.A., Lowery D.M., Ho T., Haire L.F., Yaffe M.B., Smerdon S.J. (2004). Structure and mechanism of BRCA1 BRCT domain recognition of phosphorylated BACH1 with implications for cancer. Nat. Struct. Mol. Biol..

[90] Badgujar D.C., Sawant U., Vikrant, Yadav L., Hosur M.V., Varma A.K. (2013). Preliminary crystallographic studies of BRCA1 BRCT-ABRAXAS complex. Acta Crystallogr. Sect. F Struct. Biol. Cryst. Commun..

[91] Manke I.A. (2003). BRCT Repeats As Phosphopeptide-Binding Modules Involved in Protein Targeting. Science.

[92] Vauquelin G., Charlton S.J. (2013). Exploring avidity: Understanding the potential gains in functional affinity and target residence time of bivalent and heterobivalent ligands. Br. J. Pharmacol..

[93] Hunkeler M., Hagmann A., Stuttfeld E., Chami M., Guri Y., Stahlberg H., Maier T. (2018). Structural basis for regulation of human acetyl-CoA carboxylase. Nature.

[94] Sartori A.A., Lukas C., Coates J., Mistrik M., Fu S., Bartek J., Baer R., Lukas J., Jackson S.P. (2007). Human CtIP promotes DNA end resection. Nature.

[95] Wu Q., Jubb H., Blundell T.L. (2015). Phosphopeptide interactions with BRCA1 BRCT domains: More than just a motif. Prog. Biophys. Mol. Biol..

[96] Castillo A., Paul A., Sun B., Huang T.H., Wang Y., Yazinski S.A., Tyler J., Li L., You M.J., Zou L. (2014). The BRCA1-Interacting Protein Abraxas Is Required for Genomic Stability and Tumor Suppression. Cell Rep..

[97] Wu J., Liu C., Chen J., Yu X. (2012). RAP80 Protein Is Important for Genomic Stability and Is Required for Stabilizing BRCA1-A Complex at DNA Damage Sites in Vivo. J. Biol. Chem..

[98] Xiao L., Lee K.K.H. (2016). BRE facilitates skeletal muscle regeneration by promoting satellite cell motility and differentiation. Biol. Open.

[99] Yin Z., Menendez D., Resnick M.A., French J.E., Janardhan K.S., Jetten A.M. (2012). RAP80 Is Critical in Maintaining Genomic Stability and Suppressing Tumor Development. Cancer Res..

[100] Vikrant, Kumar R., Yadav L.R., Nakhwa P., Waghmare S.K., Goyal P., Varma A.K. (2013). Structural and Functional Implication of RAP80 ΔGlu81 Mutation. PLoS ONE.

[101] Nikkilä J., Coleman K.A., Morrissey D., Pylkas K., Erkko H., Messick T.E., Karppinen S.M., Amelina A., Winqvist R., Greenberg R.A. (2009). Familial breast cancer screening reveals an alteration in the RAP80 UIM domain that impairs DNA damage response function. Oncogene.

[102] Chen C.C., Feng W., Lim P.X., Kass E.M., Jasin M. (2018). Homology-Directed Repair and the Role of BRCA1, BRCA2, and Related Proteins in Genome Integrity and Cancer. Annu. Rev. Cancer Biol..

[103] Kass E.M., Lim P.X., Helgadottir H.R., Moynahan M.E., Jasin M. (2016). Robust homology-directed repair within mouse mammary tissue is not specifically affected by Brca2 mutation. Nat. Commun..

[104] Miskinyte S., Butler M.G., Hervé D., Sarret C., Nicolino M., Petralia J.D., Bergametti F., Arnould M., Pham V.N., Gore A.V. (2011). Loss of BRCC3 deubiquitinating enzyme leads to abnormal angiogenesis and is associated with syndromic moyamoya. Am. J. Hum. Genet..

[105] Forbes S.A., Beare D., Gunasekaran P., Leung K., Bindal N., Boutselakis H., Ding M., Bamford S., Cole C., Ward S. (2015). COSMIC: Exploring the world’s knowledge of somatic mutations in human cancer. Nucleic Acids Res..

[106] Jin G., Mao X., Qiao Z., Chen B., Jin F. (2019). RAP80 expression in breast cancer and its relationship with apoptosis in breast cancer cells. OncoTargets Ther..

[107] Bolton K.L., Tyrer J., Song H., Ramus S.J., Notaridou M., Jones C., Sher T., The Australian Ovarian Cancer Study Group, The Australian Cancer Study (Ovarian Cancer), On behalf of the Ovarian Cancer Association Consortium (2010). Common variants at 19p13 are associated with susceptibility to ovarian cancer. Nat. Genet..

[108] Noordermeer S.M., Wennemers M., Bergevoet S.M., Heijden A., Tönnissen E., Sweep F.C.G.J., Jansen J.H., Span P.N., Reijden B.A. (2012). Expression of the BRCA1 complex member BRE predicts disease free survival in breast cancer. Breast Cancer Res. Treat..

[109] Noordermeer S.M., Monteferrario D., Sanders M.A., Bullinger L., Jansen J.H., van der Reijden B.A. (2012). Improved classification of MLL-AF9-positive acute myeloid leukemia patients based on BRE and EVI1 expression. Blood.

[110] Noordermeer S.M., Sanders M.A., Gilissen C., Tonnissen E., van der Heijden A., Dohner K., Bullinger L., Jansen J.H., Valk P.J.M., van der Reijden B.A. (2011). High BRE expression predicts favorable outcome in adult acute myeloid leukemia, in particular among MLL-AF9-positive patients. Blood.

[111] Balgobind B.V., Zwaan C.M., Reinhardt D., Arentsen-Peters T.J.C.M., Hollink I.H.I.M., de Haas V., Kaspers G.J.L., de Bont E.S.J.M., Baruchel A., Stary J. (2010). leu2010211a. Leukemia.

[112] Katlinski K.V., Gui J., Katlinskaya Y.V., Ortiz A., Chakraborty R., Bhattacharya S., Carbone C.J., Beiting D.P., Girondo M.A., Peck A.R. (2017). Inactivation of Interferon Receptor Promotes the Establishment of Immune Privileged Tumor Microenvironment. Cancer Cell.

[113] Kim D., Fiske B.P., Birsoy K., Freinkman E., Kami K., Possemato R.L., Chudnovsky Y., Pacold M.E., Chen W.W., Cantor J.R. (2015). SHMT2 drives glioma cell survival in ischaemia but imposes a dependence on glycine clearance. Nature.

[114] Semenza G.L. (2016). Hypoxia-inducible factors: Coupling glucose metabolism and redox regulation with induction of the breast cancer stem cell phenotype. EMBO J..

[115] Kurdekar V., Giridharan S., Subbarao J., Nijaguna M.B., Periasamy J., Boggaram S., Shivange A.V., Sadasivam G., Padigaru M., Potluri V. (2019). Structure-Guided Synthesis and Evaluation of Small-Molecule Inhibitors Targeting Protein–Protein Interactions of BRCA1 tBRCT Domain. ChemMedChem.

[116] Periasamy J., Kurdekar V., Jasti S., Nijaguna M.B., Boggaram S., Hurakadli M.A., Raina D., Kurup L.M., Chintha C., Manjunath K. (2018). Targeting Phosphopeptide Recognition by the Human BRCA1 Tandem BRCT Domain to Interrupt BRCA1-Dependent Signaling. Cell Chem. Biol..

[117] Northall S., Ivančić-Baće I., Soultanas P., Bolt E. (2016). Remodeling and Control of Homologous Recombination by DNA Helicases and Translocases that Target Recombinases and Synapsis. Genes.

[118] Ye L., Wang C., Hong L., Sun N., Chen D., Chen S., Han F. (2018). Programmable DNA repair with CRISPRa/i enhanced homology-directed repair efficiency with a single Cas9. Cell Discov..

[119] Fuchs S.Y. (2013). Hope and Fear for Interferon: The Receptor-Centric Outlook on the Future of Interferon Therapy. J. Interferon Cytokine Res..

[120] Sowa M.E., Bennett E.J., Gygi S.P., Harper J.W. (2009). Defining the Human Deubiquitinating Enzyme Interaction Landscape. Cell.

